# Data relating to maternal fish consumption, methylmercury exposure, and early child neurodevelopment in the traditional living of Western Amazonians

**DOI:** 10.1016/j.dib.2019.104153

**Published:** 2019-06-19

**Authors:** Rejane C. Marques, José G. Dórea, Monica P.L. Cunha, Thayssa C.S. Bello, José V.E. Bernardi, Olaf Malm

**Affiliations:** a Universidade Federal do Rio de Janeiro, Brazil; b Universidade de Brasilia, Brazil; c Universidade Federal de Rondonia, Brazil

**Keywords:** Child development, Fish, Methylmercury, Prenatal exposure, Hair mercury

## Abstract

This data paper includes information of a cohort organized to study the health, nutrition, and development of Amazonian children [1]. Child development were evaluated by trained nurses and psychologists with the Bayley Scales of Infant Development (at 24 months), the Stanford-Binet Intelligence Scale (at 60 months) and also with questionnaires administered by trained interviewers to the mothers. Maternal food questionnaires were used to estimate fish consumption and the associations between levels of prenatal and postnatal hair mercury (from mothers and children) and scores of neurodevelopment.

**Table:** Specifications table

Subject area	Human Biology
More specific subject area	Child Development and Growth.
Type of data	Table
How data was acquired	Longitudinal cohort study, questionnaire data (supplemental file); biological assessment
Data format	Edited, raw data are provided as a supplemental file
Experimental factors	Maternal questionnaires (supplemental file) and hair Hg concentrations
Experimental features	Mean neurodevelopment scores associated with maternal and child prenatal and postnatal Hg exposures.
Data source location	Universidade Federal do Rio de Janeiro, Rio de Janeiro, Brazil.
Data accessibility	Data are with in this article, raw data (excel file) are provided as a supplemental file.
Related research articles	[1] Marques R.C., Bernardi, J.V.E., Dórea J.G., Brandao K.G., Bueno L., Leão R.S., Malm O. Fish Consumption during Pregnancy, Mercury Transfer, and Birth Weight along the Madeira River Basin in Amazonia. International Journal of Environmental Research and Public Health, v. 10, p. 2150–2163, 2013a. https://doi.org/10.3390/ijerph10062150

**Table:** 

**Value of the Data** • The dataset comprises longitudinal information on a large sample (n = 1433) of children living in defined areas of the Rio Madeira whose growth and neurodevelopment were monitored over five years. • This set of data is key for early identification of adverse effects of environmental mercury exposures. • The data allow analyses of family socioeconomic indicators and their associations with children's growth and neurodevelopment. • The dataset can be used as a reference in children's environmental health research, because of the wholeness of the sample.

## 1 Data

In this article, we describe data on maternal fish consumption, levels of prenatal and postnatal hair mercury, and child development; dataset were summarized in Table 1, Table 2, Table 3 (raw data are provided as a supplemental file). The dataset was designed to answer the basic questions related to fish consumption (and methylmercury exposure) during both prenatal and postnatal life and also to assess their effects on children's growth and development. After contacting 1668 pregnant mothers living along 733 km of the Madeira River Basin we enrolled 1433 volunteers. After delivery, during regular visits (6, 12, 24, and 60 months) we applied questionnaires (see supplemental file), took anthropometric measurements to make a comprehensive evaluation of health, growth and neurodevelopment of their children [1]. The study was approved by the Ethics Committee for Studies in Humans of the Federal University of Rondonia (Of. 001–07/CEP/NUSAU).

**Table 1:** Publications using the questionnaire data, measurements of child development and growth.

Authors	Outcomes	Environment	Results
Marques et al., 2013a [1]	Pregnancy	Birth Weight	Gestational age and maternal education are the only variables positively affecting birth weight.
Marques et al., 2016c [2]	24 and 60 months	Birth environment (home vs hospital) and associated perinatal Hg exposures	Comparing birth environment (hospital *vs* home) revealed significant differences in maternal Hg exposure but not in child neurodevelopment.
Marques et al., 2016b [8]	24 months	Prolonged breastfeeding and mercury exposure	Hair-Hg decreased in mothers while increased in children at 24 months. Both, fish consumption and maternal education have opposing effects on Hg exposure.
Cunha et al., 2018 [9]	0, 6, 24, 59 months	Anthropometric Indices and Maternal Fish Intake	Longer duration of breastfeeding and higher maternal schooling favored weight-for-height ratio in children. Both, maternal hemoglobin concentration and age positively influenced children anthropometric indices; however, maternal fish consumption *per se* had no significant effect on children's growth.
Marques et al., 2015 [10]	6 and 24 months	Hg exposure (during pregnancy and in postnatal life) in an open-pit tin-ore mining environment	In a tin-ore mining environment neurodevelopment was weakly associated prenatal Hg exposure with sex difference; boys showed more sensitivity to delays in BSID scores.
Marques et al., 2016a [11]	24 months	Age of walking and age of talking, and the Bayley Scale of Infant Development (BSID)	The increase in neurodevelopmental (BSID) delays is a plausible form of neurotoxicity provoked by Hg exposures.
Marques et al., 2013b [12]	6 months	Exclusive breastfeeding	The used statistical model (linear regression) showed that maternal hair-Hg was a good and significant predictor of newborn hair-Hg, thus making it a reliable indicator of fetal exposure. Sex seems to influence Hg metabolism in newborns.

**Table 2:** Summary of variables related to prenatal mercury exposure (fish consumption) and child development.

Neuro-outcomes	N	Median	Min	Max	Mean ± SD
**Age at walking, m**					
All children	1433	13	10	24	13.89 ± 2.72
Mother did not eat fish	80	13	10	22	13.39 ± 2.48
Mother ate fish	1353	13	10	24	13.92 ± 2.73
**Age at talking, m**					
All children	1433	14	10	27	14.05 ± 2.64
Mother did not eat fish	80	14	10	22	14.21 ± 2.50
Mother ate fish	1353	14	10	27	14.04 ± 2.65
**MDI, 24m**					
All children	1433	95	55	125	94.10 ± 15.52
Mother did not eat fish	80	90	55	115	87.97 ± 14.18
Mother ate fish	1353	95	55	115	92.03 ± 14.04
**PDI, 24m**					
All children	1433	100	55	135	97.84 ± 12.63
Mother did not eat fish	80	91	57	115	90.05 ± 12.79
Mother ate fish	1353	95	55	115	91.92 ± 14.17
*5 years (Stanford-Binet)*					
**Fluid Reasoning**					
All children	1425	88	45	135	86.20 ± 16.45
Mother did not eat fish	80	85.5	53	118	84.19 ± 15.92
Mother ate fish	1345	88	45	135	86.32 ± 16.47
**Knowledge**					
All children	1425	87	10	132	86.02 ± 15.75
Mother did not eat fish	80	83	53	119	84.39 ± 15.53
Mother ate fish	1345	87	10	132	86.11 ± 15.76
**Quantitative reasoning**					
All children	1425	89	45	141	87.60 ± 15.65
Mother did not eat fish	80	87	58	135	87.83 ± 14.13
Mother ate fish	1345	89	45	141	87.59 ± 15.74
**Visual-Spatial Processing**					
All children	1425	90	46	134	88.32 ± 15.59
Mother did not eat fish	80	85.5	54	126	85.19 ± 15.90
Mother ate fish	1345	90	46	134	88.51 ± 15.55
**Working Memory**					
All children	1425	93	45	137	91.45 ± 15.36
Mother did not eat fish	80	97	57	130	93.76 ± 14.09
Mother ate fish	1345	93	45	137	91.31 ± 15.42
**NVIQ**					
All children	1425	90	56	137	89.70 ± 11.25
Mother did not eat fish	80	88	56	117	88.83 ± 12.23
Mother ate fish	1345	90	58	137	89.76 ± 11.19
**VIQ**					
All children	1425	91	55	132	91.62 ± 10.19
Mother did not eat fish	80	91.5	65	115	90.91 ± 10.28
Mother ate fish	1345	91	55	132	91.66 ± 10.18
**FSIQ**					
All children	1425	90	60	133	90.38 ± 10.08
Mother did not eat fish	80	90	60	112	89.55 ± 10.64
Mother ate fish	1345	90	60	133	90.43 ± 10.04

m = months; MDI = mental development index; PDI = psychomotor development index; NVIQ Nonverbal intelligence quotient; VIQ = verbal intelligence quotient; FSIQ = full scale intelligence quotient.

**Table 3:** Maternal and children biodata related to the mercury and anthropometric indices (Z-score).

	N	Median	Min	Max	Mean ± SD
**Hair-Hg (μg·g**^**−1**^**)**					
*Children*					
HgRN	1344*	1.45	0.09	18.53	2.05 ± 1.85
HgT, 6 m	1433	2.02	0.15	18.95	2.56 ± 1.97
HgT, 2y	1433	2.94	0.21	23.24	3.41 ± 2.37
HgT, 5y	1425*	3.06	0.4	20.37	3.62 ± 2.45
*Mother*					
HgM	1433	5.81	0.73	130.72	8.47 ± 10.12
HgM, 6 m	1433	5	0.5	125.21	7.80 ± 10.02
HgM, 2y	1433	5.59	0.49	129.15	8.38 ± 10.31
HgM, 5y	1425*	5.64	0.55	146.87	8.64 ± 11.11
**Anthropometric indices (Z-score)**					
*Birth*	1433				
WHZ		−1.02	−4.34	2.75	−1.05 ± 0.92
HAZ		0.59	−3.64	5.56	0.67 ± 1.41
WAZ		−0.2	−3.19	5.06	−0.14 ± 0.97
BAZ		−0.79	−3.6	2.69	−0.76 ± 0.75
HCZ		−0.32	−3.01	3.9	−0.18 ± 0.95
BMI		12.4	9.6	17.5	12.49 ± 0.89
**6 months**	1433				
WHZ		−1.16	−3.94	2.43	−1.07 ± 0.77
HAZ		0.53	−3.13	2.48	0.24 ± 1.07
WAZ		−0.72	−2.92	1.45	−0.73 ± 0.71
BAZ		−1.25	−3.96	2.32	−1.17 ± 0.75
HCZ		−0.16	−2.75	2.22	0.11 ± 0.88
BMI		15.4	12.6	20.9	15.54 ± 0.97
**24 months**	1433				
WHZ		0.43	−4.11	4.78	0.40 ± 1.31
HAZ		−0.38	−2.23	4.86	−0.32 ± 0.87
WAZ		0.25	−2.25	3.22	0.17 ± 0.89
BAZ		0.53	−4.83	4.94	0.48 ± 1.40
HCZ		−0.2	−2.46	1.87	−0.22 ± 0.78
BMI		16.5	11.2	24	16.62 ± 1.89
**59 months**	1425*				
WHZ		−0.38	−2.45	3.48	−0.17 ± 0.98
HAZ		−0.84	−1.94	1.23	−0.71 ± 0.58
WAZ		−0.77	−2.42	2.29	−0.51 ± 0.94
BAZ		−0.35	−2.37	3.31	−0.13 ± 0.97
HCZ		−0.51	−1.83	1.6	−0.41 ± 0.57
BMI		14.8	12.5	20.9	15.14 ± 1.42

N*: differences in sample size were due to insufficient hair for analysis and/or children moved. WHZ = Weight-for-length and weight-for-height z-score; WAZ = Weight-for-age z-score; HAZ = Length or height-for-age z-score; HCZ = Head circumference-for-age z-score; BAZ = body mass index for age z-score; BMI = Body mass index; Hg = mercury; m = months; y = years.

## 2 Experimental design, materials, and methods

### 2.1 Questionnaire assessments

During programmed visits, a questionnaire was applied to collect socioeconomic data and information on habituation of family fish consumption [2]. Socioeconomic data containing demographic information, family size and income, level of maternal educational level, and breast-feeding practices were collected by trained interviewers using appropriate questionnaires. During visits, age of walking and age of talking were also collected.

### 2.2 Anthropometric measurements

Infants and children anthropometry were taken by experienced nurses; babies and infants dressed in light clothing were measured in recumbent position (height/length and weight) respectively with a stadiometer (0.1 cm) and with an electronic scale (nearest 0.1 kg). Older children had measurements taken in standing position, barefoot and dressed in underwear [2], [3]. Age of children, weight and height values were transformed into Z-scores for weight-for-age (W/A-Z), height-for-age (H/A-Z) and weight-for-height (W/H-Z), using WHO Anthro (version 3.2.2., January 2011).

### 2.3 Neurodevelopment measurements

Childhood neurodevelopment was assessed through milestone achievement (age of talking and age of walking) and with the Bayley Scales for Infant Development-BSIDII [4] applied at the ages of 6 and 24 months; at the age of 60 months the intelligence quotient (IQ) was assessed by the Stanford-Binet Intelligence Scale [5]. These tests (BSIDII as well as IQ) were applied in the quiet and familiar home environment of the children by qualified and experienced psychologists. The BSIDII measure cognitive, language, motor, social, emotional, and adaptive behavior; these make up scores of the Mental Developmental Index (MDI) and Psychomotor Developmental Index (PDI). The MDI score provides an index for general cognitive development (including tap memory, habituation, problem solving, early number concepts, generalization, classification, vocalizations, language, and social skills). The PDI score provides an index for motor development (fine and gross motor skills, e.g., rolling, crawling, creeping, sitting, standing, walking, running, jumping, apprehension, use of writing implements, and imitation of hand movements). The integrated test results in scores indicate global development: severe delays (score <69) and mild delays (scores 70–84) delays, normal limits (scores 85–114) of development, and accelerated performance (score >115) [4].

The Stanford-Binet Intelligence Scale (SB5) is designed to assess both abilities and aptitudes; this test encompasses five factors: Fluid reasoning, knowledge, quantitative reasoning, visual-spatial processing, and working memory. In each factor there are two subsets: one verbal subtest and one nonverbal subtest; the test outcomes are combined in a composite intelligence quotient (IQ). Therefore, the SB5 ends up with 10 subtest scores (Mean (SD) = 10 (3), range 1–19) that can be combined to create scores (factor and composite). The five subtests (containing nonverbal and five verbal) are combined into two domain composite scores: the Nonverbal IQ (NVIQ) and the Verbal IQ (VIQ); each domain contains one subtest from the five factors. All combined 10 subtests yield the Full Scale IQ (FSIQ) composite score. The FSIQ measures the general ability to solve problems, reason, and adapt to the cognitive demands of the environment; thus reflecting the five major facets of intelligence: stored information, reasoning, visualization, memory, and the ability to solve novel problems. The FSIQ is used to predict long-term educational attainment. The indexes and the composite scores have a population mean of 100 and a standard deviation of 15 [5], [6].

### 2.4 Hair collection and mercury determination

Head hair samples were taken from mothers and all children during home visits and anthropometric measurements (measuring height and weight). Hair samples were cut from both mother and newborn from the back of the head close to the scalp (always in the same occipital area) using stainless steel scissors. The hair samples were bundled together, properly labeled, stored in an envelope, and taken to analysis. Hair processing and total Hg determination were done according to the analytical methods used in the Radioisotopes Laboratory of the Federal University of Rio de Janeiro [1], [7]. The analytical method starts with sample washing with EDTA (0.01%), and digestion (5 mL of HNO3:H2SO4 (1:1) and 4 mL of 5% KMnO4) followed by reduction to elemental Hg vapor. Total Hg determination was done by cold vapor atomic absorption spectrometry with a flow injection system/FIMS (CV-AAS; Perkin-Elmer-FIMS 400, Ueberlingen, Germany).

## 3 Publications

Publications using data from the questionnaire, measurements of child growth and neurodevelopment and are summarized in Table 1. Positive associations were found for prenatal fish intake [8], maternal education [1], [9] and breastfeeding [8], [9]*,* and negative associations between sex (boys) and neurodevelopment [10]. In regard to Hg exposure we show that neurodevelopmental (BSID) delays are a plausible form of neurotoxicity provoked by Hg exposures [11] and that sex may influence Hg metabolism in newborns [12]. Besides, HHg increased in breastfed children and decreased in mothers at 24 months [8].
